# Assessing HIV risk and the social and behavioural characteristics of gay and bisexual men who have recently migrated to Australia: an analysis of national, behavioural surveillance data 2019–2021

**DOI:** 10.1002/jia2.26204

**Published:** 2024-01-09

**Authors:** Simin Yu, Benjamin R. Bavinton, Curtis Chan, James MacGibbon, Limin Mao, Daniel Vujcich, Timothy R. Broady, Martin Holt

**Affiliations:** ^1^ Centre for Social Research in Health University of New South Wales Sydney New South Wales Australia; ^2^ The Kirby Institute University of New South Wales Sydney New South Wales Australia; ^3^ School of Population Health Curtin University Perth Western Australia Australia

**Keywords:** gay and bisexual men, HIV prevention, HIV testing, men who have sex with men, migrant, pre‐exposure prophylaxis

## Abstract

**Introduction:**

Overseas‐born gay and bisexual men (GBM) are overrepresented in HIV diagnoses in Australia. We assessed social and sexual behaviours, and the use of HIV prevention and testing, by region of birth and length of residence in Australia. We sought to identify similarities and differences between recently arrived and non‐recently arrived GBM from non‐English‐speaking countries to improve targeting and engagement with HIV testing and prevention.

**Methods:**

Data were collected in national repeated, behavioural surveillance surveys conducted across Australia during 2019–2021. Logistic regression was used to identify factors that differentiated between recently arrived (<2 years) and non‐recently arrived (≥2 years in Australia) GBM from non‐English‐speaking countries.

**Results:**

Among 24,707 participants in 2019–21, 2811 (11.4%) were from high‐income English‐speaking countries, 714 (2.9%) were recently arrived overseas‐born GBM and 3833 (15.5%) were non‐recently arrived migrants. Recently arrived GBM were most likely to be born in Asia (36.1%) and Europe (21.1%). Compared with non‐recently arrived GBM, recently arrived GBM from non‐English‐speaking countries were younger (aOR = 0.95, 95% CI = 0.94–0.96, *p*<0.001), more likely to be students (aOR = 1.43, 95% CI = 1.11–1.85, *p* = 0.005), less likely to be in full‐time employment (aOR = 0.56, 95% CI = 0.46–0.69, *p* <0.001), more likely to report consistent condom use (aOR = 1.30, 95% CI = 1.01–1.66, *p* = 0.039), but had lower awareness (aOR = 0.62, 95% CI = 0.48–0.80, *p*<0.001) and use of pre‐exposure prophylaxis (PrEP) (22.8%, vs. 32.3%, χ^2^(1, 4185) = 23.78, *p*<0.001), and similar levels of casual sex with a risk of HIV acquisition or transmission (aOR = 1.29, 95% CI = 0.98–1.69, *p* = 0.066). Recently arrived GBM reported similar levels of lifetime HIV testing (aOR = 0.97, 95% CI = 0.54–1.74, *p* = 0.915) and recent HIV testing (OR = 1.03, 95% CI = 0.86–1.22, *p* = 0.779), but were much less likely to have tested at general practitioners (aOR = 0.53, 95% CI = 0.41–0.68, *p*<0.001) and more likely to report testing at hospitals (aOR = 3.35, 95% CI = 2.53–4.43, *p*<0.001), at home (aOR = 2.85, 95% CI = 1.63–4.99, *p*<0.001), or community‐based services (aOR = 1.36, 95% CI = 1.01–1.84, *p* = 0.043).

**Conclusions:**

Recently arrived GBM from non‐English‐speaking countries reported similar levels of risk of HIV acquisition to longer‐term residents in Australia, but lower levels of PrEP awareness and use, and more reliance on HIV testing services which are free or low cost. It is necessary to enhance access to HIV testing and prevention among recently arrived GBM in Australia.

## 1 INTRODUCTION

In 2020, 29.8% of Australia's population was born overseas and over 7.6 million migrants were living in Australia [1]. In 2019, there were 2.43 million temporary migrants in Australia [2], many of whom were international students [3].

Migrants are a priority for HIV prevention in Australia, which has an HIV epidemic concentrated among gay and bisexual men (GBM) [4]. During 2012–19, annual HIV diagnoses decreased by 43% among Australian‐born GBM, but diagnoses rose 54% among overseas‐born men, particularly from Asia [5, 6, 7].

Length of residence in Australia may affect migrants’ sexual behaviour and use of HIV prevention [6, 7, 8]. Recently arrived GBM who have been diagnosed with HIV report less access to and use of prevention methods prior to their diagnosis, particularly pre‐exposure prophylaxis (PrEP) [6]. Known barriers included language fluency [8, 9], lower incomes [10], cultural and religious factors [11, 12], a perception that HIV is not a public health concern in Australia [9], fear of stigma or discrimination [13, 14, 15, 16, 17] and poorer knowledge of effective HIV prevention strategies [6, 11, 12]. Medicare, Australia's national health insurance scheme, is not available to most temporary residents, who may need to pay for private health insurance and to attend health services [18]. These issues are particularly acute among newer, recently arrived migrants from non‐English‐speaking countries [8, 9, 15].

Previous research has been conducted with overseas‐born GBM in Australia, utilizing small clinic‐based samples [6, 7, 8, 15, 19]. These studies showed that many recent arrivals were international students who spoke a language other than English at home [6, 8, 15], did not have Medicare coverage [6, 8], and had poorer knowledge about and were less likely to access HIV testing than Australian‐born GBM [6, 8, 15]. The current analysis used a national, community‐based sample of GBM, to compare the characteristics of recently and non‐recently arrived overseas‐born GBM, in order to improve the targeting of HIV prevention and testing.

## 2 METHODS

### 2.1 Participants and recruitment

Data were collected from the Gay Community Periodic Surveys [20, 21], repeated cross‐sectional surveys that recruit GBM from gay community events, venues, and online in seven states and territories. Time‐location sampling is used for venue and event‐based recruitment. Paper questionnaires (in English) are distributed and collected by trained staff. Online recruitment is driven by paid Facebook advertising. The online questionnaire is available in English, simplified and traditional Chinese, Indonesian, Portuguese, Spanish, Thai and Vietnamese.

Participants were eligible if they were ≥16 years old (online recruitment) or ≥18 years old (in‐person recruitment), identified as male (cisgender or transgender), as gay or bisexual, or had sex with another man in the past 5 years and lived in Australia. Ethical approval was received from the University of New South Wales (HC 180903) and two community organizations (ACON and Thorne Harbour Health). For in‐person recruitment, participants are provided study information by a recruiter, and are provided with a questionnaire to self‐complete, after giving verbal consent. For online recruitment, participants read the study information online, and access the online questionnaire after giving electronic consent.

### 2.2 Measures

Migration/residency status was identified using participants’ country of birth and length of time living in Australia. Countries of birth were categorized into eight groups: Africa, Australia, other high‐income English‐speaking countries, Asia, Europe, Central and South America, Oceania and the Middle East. Migrants living in Australia for less than 2 years were considered recently arrived, and for 2 or more years non‐recently arrived.

Demographic variables included age, sexual identity, student status, education, employment and state of residence. Gay social engagement (GSE) was measured with two questions: how many of your friends are gay men (scored 0–4) and how much of your free time is spent with gay men (scored 0–3). These scores were summed with participants who scored ≤3 categorized as having low GSE and participants scoring 4–7 high GSE [22]. Sexual behaviour included the number of recent male sex partners and any group sex (in the past 6 months). Sexual behaviour with casual male partners in the previous 6 months was categorized into five groups: no casual partners; casual partners but no anal intercourse; using condoms consistently for anal intercourse; having condomless sex but being HIV negative and on PrEP, or HIV positive, on treatment and having an undetectable viral load (UVL); having condomless sex and being HIV negative or untested and not on PrEP, or being HIV positive, not on treatment or having a detectable viral load (DVL). Participants in groups 1–4 were classified as reporting casual sex without a risk of HIV acquisition or transmission and group 5 as reporting casual sex with a risk of HIV acquisition or transmission [23]. Self‐reported HIV status, recent drug use, sexually transmitted infection (STI) testing, HIV testing frequency and locations for testing were also included.

### 2.3 Analysis

Bivariate and multivariate logistic regression models were used to compare recently arrived and non‐recently arrived overseas‐born GBM, excluding those from high‐income English‐speaking countries. Variables for which there was a statistical difference at a bivariate level of *p*<0.10 were included in a multivariable logistic regression model to identify statistically independent differences (*p*<0.05). Analysis was conducted using Stata version 16.0 (StataCorp, College Station, TX).

## 3 RESULTS

In total, 24,815 questionnaires were completed between 2019 and 2021, 24,620 in English, 195 in other languages, 12,932 (52.1%) in person and 11,883 (47.9%) online. One hundred and eight participants were excluded who did not provide a country of birth. Of the 24,707 remaining, 70.1% were born in Australia, 11.4% in high‐income English‐speaking countries and 18.6% from other countries. Participants born in Australia and high‐income English‐speaking countries were excluded.

Among 4585 GBM born in non‐English‐speaking countries, 38 did not indicate the length of residence in Australia and were also excluded. Of the final 4547 participants, 15.7% had been living in Australia for less than 2 years (recently arrived) and 84.3% had been living in Australia for 2 years or more (non‐recently arrived) (Table 1). The most common region of birth was Asia. The top five countries of birth among recently arrived GBM were Brazil, Colombia, the Philippines, China and Malaysia, while among non‐recently arrived GBM, they were Malaysia, the Philippines, South Africa, China and India.

**Table 1:** Participant characteristics and multivariate logistic regression comparing recently arrived and non‐recently arrived GBM (*N* = 4547)

	Total *N* = 4547	Non‐recently arrived *n* = 3833	Recently arrived *n* = 714	OR (95% CI)	*p*	AOR (95% CI)	*p*
Region of birth							
Asia	1936 (42.6)	1678 (43.8)	258 (36.1)	Ref.		Ref.	
Europe	734 (16.1)	583 (15.2)	151 (21.1)	1.68 (1.35−2.10)	<0.001^*^	2.67 (2.09−3.42)	<0.001^*^
Central or South America	583 (12.8)	434 (11.3)	149 (20.9)	2.23 (1.78−2.80)	<0.001^*^	2.22 (1.73−2.85)	<0.001^*^
Africa	356 (7.8)	332 (8.7)	24 (3.4)	0.47 (0.30−0.73)	0.001^*^	0.81 (0.50−1.32)	0.396
Oceania	85 (1.9)	80 (2.1)	5 (0.7)	0.41 (0.16−1.01)	0.053	0.59 (0.24−1.44)	0.248
Middle East	189 (4.2)	163 (4.3)	26 (3.6)	1.04 (0.67−1.60)	0.868	1.28 (0.79−2.06)	0.315
Overseas—unspecified	664 (14.6)	563 (14.7)	101 (14.1)	1.17 (0.91−1.50)	0.225	1.62 (1.23−2.13)	0.001^*^
Age (M/IQR)	33 (28−41)	35 (29−42)	29.0 (25−33)	0.93 (0.92−0.95)	<0.001^*^	0.95 (0.94−0.96)	<0.001^*^
Sexual identity							
Gay	3883 (85.4)	3290 (85.8)	593 (83.1)	Ref.			
Bisexual	458 (10.1)	373 (9.7)	85 (11.9)	1.26 (0.98−1.63)	0.067		
Others	206 (4.5)	170 (4.4)	36 (5.0)	1.17 (0.81−1.70)	0.393		
Student							
No	3832 (84.3)	3346 (87.3)	486 (68.1)	Ref.		Ref.	
Yes	715 (15.7)	487 (12.7)	228 (31.9)	3.22 (2.68−3.87)	<0.001^*^	1.43 (1.11−1.85)	0.005^*^
Education							
No university education	1187 (26.1)	1015 (26.5)	172 (24.1)	Ref.			
University educated	3360 (73.9)	2818 (73.5)	542 (75.9)	1.14 (0.94−1.37)			
Full‐time employment							
No	1791 (39.4)	1371 (35.8)	420 (58.8)	Ref.		Ref.	
Yes	2756 (60.6)	2462 (64.2)	294 (41.2)	0.39 (0.33−0.46)	<0.001^*^	0.56 (0.46−0.69)	<0.001^*^
State							
New South Wales	2110 (46.4)	1751 (45.7)	359 (50.3)	Ref.		Ref.	
Victoria	1395 (30.7)	1166 (30.4)	229 (32.1)	0.96 (0.80−1.15)	0.643	1.02 (0.84−1.25)	0.823
Queensland	506 (11.1)	435 (11.3)	71 (9.9)	0.80 (0.60−1.05)	0.105	0.78 (0.58−1.05)	0.102
Others	536 (11.8)	481 (12.5)	55 (7.7)	0.56 (0.41−0.75)	<0.001^*^	0.53 (0.38−0.74)	<0.001^*^
Gay social engagement							
Low	1646 (36.2)	1355 (35.4)	291 (40.8)	Ref.		Ref.	
High	2901 (63.8)	2478 (64.6)	423 (59.2)	0.79 (0.68−0.94)	0.006^*^	0.95 (0.79−1.14)	0.559
Ever been tested for HIV							
No	415 (9.1)	326 (8.5)	89 (12.5)	Ref.		Ref.	
Yes	4132 (90.9)	3507 (91.5)	625 (87.5)	0.65 (0.51−0.84)	0.001^*^	0.97 (0.54−1.74)	0.915
HIV testing (last 12 months)							
No	1415 (31.1)	1196 (31.2)	219 (30.7)	Ref.			
Yes	3132 (68.9)	2637 (68.8)	495 (69.3)	1.03 (0.86−1.22)	0.779		
Last HIV test at a GP							
No	3178 (69.9)	2555 (66.7)	623 (87.3)	Ref.		Ref.	
Yes	1369 (30.1)	1278 (33.3)	91 (12.7)	0.29 (0.23−0.37)	<0.001^*^	0.53 (0.41−0.68)	<0.001^*^
Last HIV test at a sexual health clinic							
No	2750 (60.5)	2307 (60.2)	443 (62.0)	Ref.			
Yes	1797 (39.5)	1526 (39.8)	271 (38.0)	0.92 (0.78−1.09)	0.352		
Last HIV test at a hospital							
No	4211 (92.6)	3622 (94.5)	589 (82.5)	Ref.		Ref.	
Yes	336 (7.4)	211 (5.5)	125 (17.5)	3.64 (2.87−4.62)	<0.001^*^	3.35 (2.53−4.43)	<0.001^*^
Last HIV test at home							
No	4488 (98.7)	3797 (99.1)	691 (96.8)	Ref.		Ref.	
Yes	59 (1.3)	36 (0.9)	23 (3.2)	3.51 (2.07−5396)	<0.001^*^	2.85 (1.63−4.99)	<0.001^*^
Last HIV test at community‐based service							
No	4160 (91.5)	3522 (91.9)	638 (89.4)	Ref.		Ref.	
Yes	387 (8.5)	311 (8.1)	76 (10.6)	1.35 (1.03−1.76)	0.027^*^	1.36 (1.01−1.84)	0.043^*^
HIV status							
HIV negative	3743 (82.3)	3167 (82.6)	576 (80.7)	Ref.		Ref.	
HIV positive	362 (8.0)	314 (8.2)	48 (6.7)	0.84 (0.61−1.15)	0.282	1.24 (0.88−1.75)	0.222
Unknown/untested	442 (9.7)	352 (9.2)	90 (12.6)	1.41 (1.10−1.80)	0.007^*^	0.94 (0.54−1.65)	0.831
Any STI diagnosis (last 12 months)							
No	3369 (74.1)	2833 (73.9)	536 (75.1)	Ref.			
Yes	1178 (25.9)	1000 (26.1)	178 (24.9)	0.94 (0.78−1.13)	0.516		
PrEP awareness							
Not heard of PrEP	570 (12.5)	427 (11.1)	143 (20.0)	Ref.		Ref.	
Knew PrEP was available	3977 (87.5)	3406 (88.9)	571 (80.0)	0.50 (0.41−0.62)	<0.001^*^	0.62 (0.48−0.80)	<0.001^*^
Number of male sex partners (last 6 months)							
0–10	3580 (78.7)	3016 (78.7)	564 (79.0)	Ref.			
>10	967 (21.3)	817 (21.3)	150 (21.0)	0.98 (0.81−1.19)	0.854		
Sexual behaviour with casual male partners (last 6 months)							
No casual partners	1756 (38.6)	1477 (38.5)	279 (39.1)	Ref.		Ref.	
Casual partners but no anal intercourse	427 (9.4)	370 (9.7)	57 (8.0)	0.82 (0.60−1.11)	0.193	0.92 (0.67−1.28)	0.620
Casual partners and consistent condom use	732 (16.1)	587 (15.3)	145 (20.3)	1.31 (1.05−1.63)	0.018^*^	1.30 (1.01−1.66)	0.039^*^
Condomless sex with casual partners, but on PrEP or undetectable viral load	1027 (22.6)	914 (23.8)	113 (15.8)	0.65 (0.52−0.83)	<0.001^*^	0.87 (0.66−1.14)	0.318
Condomless sex with casual partners, but not on PrEP, or not on treatment or detectable viral load	605 (13.3)	485 (12.7)	120 (16.8)	1.31 (1.03−1.66)	0.026^*^	1.29 (0.98−1.69)	0.066
Group sex (last 6 months)							
No	3097 (68.1)	2594 (67.7)	503 (70.4)	Ref.			
Yes	1450 (31.9)	1239 (32.3)	211 (29.6)	0.88 (0.74−1.05)	0.145		
Used drugs for sex (last 6 months)							
No	3863 (85.0)	3238 (84.5)	625 (87.5)	Ref.		Ref.	
Yes	684 (15.0)	595 (15.5)	89 (12.5)	0.77 (0.61−0.98)	0.036^*^	0.85 (0.65−1.11)	0.235

Abbreviations: CI, confidence interval; GP, general practitioner; M/IQR, median/interquartile rage; PrEP, pre‐exposure prophylaxis; Ref., reference; STI, sexually transmitted infection.

* *p*<0.05.

In the bivariate results, recently arrived GBM were less likely to have ever tested for HIV compared with non‐recently arrived GBM, but similar proportions had tested within the past year (Table 1). Recently arrived GBM were less likely to have recently tested at a general practitioner (GP), and more likely to have tested at a hospital, at home or a community‐based service. Recently arrived GBM were more likely to report an unknown or untested HIV status. Levels of STI diagnoses in the last 12 months were similar. Recently arrived GBM had lower PrEP awareness than non‐recently arrived GBM. Restricting the sample to non‐HIV‐positive overseas‐born GBM (*n* = 4185), recently arrived GBM were less likely than non‐recently arrived GBM to have used PrEP in the last 6 months (22.8% vs. 32.3%, χ^2^(1, 4185) = 23.78, *p*<0.001). Further restricting the sample to overseas‐born GBM who had used PrEP (*n* = 1290), recently arrived GBM were less likely than non‐recently arrived GBM to get PrEP from a pharmacy (30.9% vs. 66.0%, χ^2^(1, 1290) = 70.31, *p*<0.001), and more likely to buy it online from overseas (44.7% vs. 16.6%, χ^2^(1, 1290) = 66.50, *p*<0.001).

Recently arrived GBM were more likely to consistently use condoms with casual partners and were less likely to report condomless sex protected by PrEP or UVL (Table 1). Recently arrived GBM were more likely to report condomless sex with a risk of HIV acquisition or transmission. Of the 120 recently arrived GBM who reported condomless sex without protection from PrEP or UVL, 113 were HIV negative or untested and not on PrEP, and seven were living with HIV, not on treatment or with a DVL. Recently arrived GBM were less likely to report group sex in the last 6 months, or to use drugs for sex.

The multivariable logistic regression model (Table 1) showed that compared with non‐recently arrived GBM, recently arrived GBM were more likely to be born in Europe, or Central or South America, compared with Asia. Recently arrived GBM were younger, more likely to be students and less likely to be in full‐time employment. Recently arrived GBM were less likely to reside in the Australian Capital Territory, South Australia, Tasmania and Western Australia, compared to New South Wales. Recently arrived GBM were less likely to have last tested for HIV at a GP, and more likely to have tested at a hospital, at home or a community‐based service. Recently arrived GBM were less likely to be aware of PrEP, and more likely to report consistent condom use with casual partners. The two groups had similar levels of GSE, likelihood of ever having been tested for HIV, HIV status distribution, sex with HIV acquisition or transmission risk and recent drug use for sex.

## 4 DISCUSSION

We used a national, community‐based sample to compare recently arrived and non‐recently arrived GBM in Australia, focusing on GBM born in non‐English‐speaking countries. We found that recently arrived GBM were more likely than non‐recently arrived GBM to be younger, born in Europe, Central or South America and be students. Recently arrived GBM had lower levels of awareness and use of PrEP, echoing previous Australian research [6, 8, 19], and reported similar levels of casual sex with a risk of HIV acquisition to longer‐term residents. Recently arrived migrants were less likely to report HIV testing at services that may incur a cost, like GPs. Our research underscores the need to encourage engagement with HIV prevention and testing in Australia among recently arrived GBM from non‐English‐speaking countries.

Recently arrived GBM reported similar levels of lifetime and recent HIV testing to non‐recent migrants. However, recently arrived GBM were more likely to test at locations other than GPs, suggesting that cost and privacy may be issues. Attending GPs may incur a fee (in contrast to sexual health clinics or community services), while testing at home does not require you to ask for a test or explain why you want one. Our findings suggest that it would be useful to publicize services where HIV testing is free or low cost to recently arrived GBM, while also reassuring recently arrived GBM about the confidentiality of HIV testing in Australia [17]. As nearly one‐third of the recently arrived GBM in our sample were students, university‐based HIV testing promotion strategies also seem warranted [24, 25].

Recently arrived GBM reported higher levels of condom use than non‐recently arrived GBM, but lower PrEP awareness and use. They reported a similar level of sex with a risk of HIV acquisition as non‐recently arrived GBM. Previous Australian studies have found that overseas‐born GBM rely on condoms more than other GBM [6, 19], and may be interested in PrEP, but consider it too expensive without Medicare coverage [6, 19, 26]. In our study, recently arrived GBM who were PrEP users were more likely than non‐recent migrants to be personally importing PrEP from overseas, which can be a cheaper way to purchase it [27]. Overseas‐born people may have concerns about PrEP's side effects, effectiveness, lack of protection from STIs and stigmatizing attitudes towards PrEP and PrEP users [19, 28, 29, 30, 31, 32]. Our results suggest that recently arrived GBM should be supported to maintain condom use, if that is their preferred strategy, but there is an opportunity to promote PrEP to recently arrived GBM, if they want to use it, particularly through free or low‐cost schemes.

There are limitations to our analysis. The GBM we recruited were accessed through community settings and online networks, which means GBM who are more isolated and less socially and sexually active are likely to be underrepresented. Most participants were recruited and completed the survey in English, which excludes GBM with poorer English language skills. The questionnaire uses recall periods of 6–12 months, which may be subject to recall bias. During 2019–2021, the COVID‐19 epidemic caused restrictions on travel and migration, so temporary residents may be underrepresented in the sample. Nevertheless, our research includes the largest sample of overseas‐born GBM analysed to date in Australia, confirming that recently arrived GBM from non‐English‐speaking countries should be prioritized in HIV prevention.

## 5 CONCLUSIONS

Our analysis indicates that recently arrived GBM in Australia report similar HIV risks to non‐recently arrived GBM, more reliance on condoms and less use of PrEP. Lower levels of awareness and affordability appear to be issues for access to HIV services, like PrEP and testing at GPs. It is necessary to enhance access to testing and effective forms of HIV prevention among recently arrived GBM. A range of interventions, including those delivered through university networks, deserve to be considered.

## COMPETING INTERESTS

In the last 3 years, BRB has received payment or honoraria from Gilead Sciences, ViiV Healthcare and FHI 360, and received unrestricted research grants from Gilead Sciences and ViiV Healthcare. DV has received payment made to his institution from Gilead for participation in the HIV Migrant Roundtable. TRB has received payment from Gilead to provide expert feedback at an online workshopping event. For the remaining authors, no conflicts were declared.

## AUTHORS’ CONTRIBUTIONS

SY, BRB, TRB, CC and MH contributed to the conceptualization of the topic. SY, CC and TRB contributed to the data curation. SY analysed the data and drafted the paper. JM and TRB helped with data validation. Funding was acquired by SY, BRB, LM, DV, TRB and MH. All authors read, commented on and approved the final manuscript.

## FUNDING

The study was funded by the Australian Government Department of Health, China Scholarship Council (No. 202206370055), National Health and Medical Research Council Partnership Project (GNT2002625) and State/Territory Health Departments.

## Data Availability

Deidentified data and syntax used in this analysis will be shared to researchers after a request to the corresponding author.
